# Gastrointestinal involvement revealing Henoch Schonlein purpura in adults: Report of three cases and review of the literature

**DOI:** 10.1186/1755-7682-4-31

**Published:** 2011-09-29

**Authors:** Amira Hamzaoui, Wissem Melki, Olfa Harzallah, Leila Njim, Rim Klii, Silvia Mahjoub

**Affiliations:** 1 Department of Internal Medicine, Fattouma Bourguiba Hospital, Monastir, Tunisia; 2 Department of Hepatogastroenterology, Fattouma Bourguiba Hospital, Monastir, Tunisia; 3 Departement of Anatomopathology, Fattouma Bourguiba Hospital, Monastir, Tunisia

**Keywords:** Adult Henoch Schönlein purpura, Gastrointestinal involvement

## Abstract

The diagnosis of Henoch-Schönlein purpura (HSP) is difficult, especially when abdominal symptoms precede cutaneous lesions.

We report three cases of adult HSP revealed by gastrointestinal (GI) involvement.

## Introduction

Henoch Schonlein purpura (HSP) is a leucocytoclastic vasculitis involving small vessel with deposition of immune Ig A complexes. HSP primarily affects children, (approximately 15 cases/100.000 per year) [1]. It is less common among adults. Clinically, HSP is characterized by palpable purpura, arthritis, renal and gastrointestinal involvement. Among adults, the incidence of HSP, the clinical features and the severity seems to be different compared to children [2]. Except for potential intestinal complications, its prognosis is usually excellent. However, nephritis is another complication that might have less prognosis. We report three cases of adult-onset HSP revealed by gastrointestinal (GI) involvements.

## Case reports

Over a period of 2 years (2009-2010), three patients with adult-onset HSP were admitted to Department of Internal Medicine. The clinical profiles, along with laboratory and radiological data, of the three patients are shown in Table 1. GI involvements were the first symptoms in the 3 cases. The delay between the GI involvement and the others symptoms is shown in table 1. Skin biopsy showed leucocytoclastic vasculitis with Ig A and C3 deposits in direct immunofluorescence (DIF) in the 3 cases.

**Table 1:** Clinical, demographic and laboratory findings of the 3 patients

Feature	Case 1	Case 2	Case 3
Age (years)/Gender	62/F	20/M	66/M
GI involvment	Hematemesis/abdominal pain	Severe abdominal pain/vomitting	Colicky abdominal pain/vomiting.
Skin rash	Lower limb and abdomen	Lower limb	Lower limb
Joints involved	Joint tenderness	Arthritis of the knee and ankles	arthritis of the ankle
Delay between GI involvement and other symptoms	3 weeks	4 weeks	2 weeks
Haemoglobin (g/dL)	10.3	15.6	14.7
Total leucocyte count (/μL)	13.000	10.400	12.200
Platelet count (/μL)	343.000	360.000	371.000
ESR (mm/1st hour)	45	22	37
Urine microscopy	Microscopc hematuria	Normal	Normal
24-hour urinary proteins	3.3 gr	Nil	Nil
Renal function	Normal	Normal	Normal
occult blood stool tests	positive	Not done	negative
Colonoscopy	colic poliposis with moderate dysplasia on the biopsy.	Not done	Not done
CT of the abdomen	Not done	Normal	Not done
Skin biopsy	Leucocytoclastic vasculitis DIF: Ig A	Leucocytoclastic vasculitis DIF: Ig A/C3	Leucocytoclastic vasculitis DIF: Ig A/C3
Oesogastroduodenal endoscopy/Biopsy	Cardial ulcer with stigmata of bleeding and purpuric lesions of the bulb and the duodenum/presence of leucocytoclastic vasculitis with intestinal metaplasia and Hp gastritis	Antrite congestive with purpuric lesions of the bulb and the duodenum/presence of leucocytoclastic vasculitis with chronic and moderately active Hp antral gastritis	Purpuric lesions of the bulb and ulcers of the DI and DII portion of the bulb/erosive duodenitis and leucocytoclastic vasculitis 1
Kidney biopsy	Extracapillar glomerulonephritis with Ig A depositis in DIF	Not done	Not done
treatment	Methylprednisolone pulse (1 g/day × 3 days) + oral prednisolone associated with cyclophosphamide pulse	Colchicine^®^ PPI	Colchicine^®^ PPI
Outcome	Complete recovery	Complete recovery	Complete recovery

ESR: erythrocyte sedimentation rate; HPE: histopathology; DIF: direct immunofluorescence; GI: Gastrointestial ; PPI: proton pump inhibitors; Hp: Helicobacter pylori

**Figure 1:**
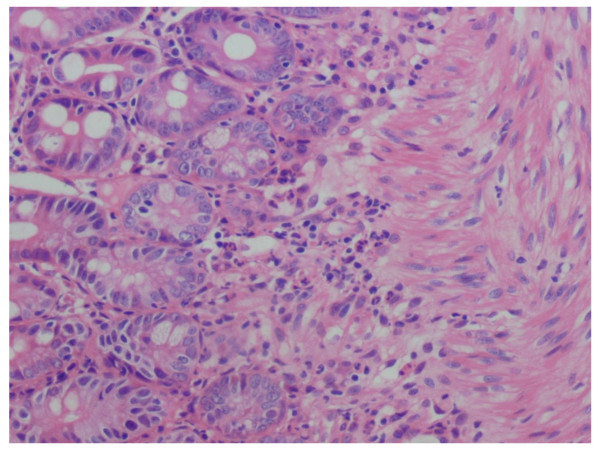
**Leucocytoclasic vasculitis of the duodenum**.

The oesogastroduodenal endoscopy with biopsy showed the presence of helicobacter pylori in case 1 and 2. The rapid urease tests for Helicobacter pylori was not done.

In the first case, the colonoscopy showed the presence of colic poliposis with moderate dysplasia on the biopsy. Tumor markers (ACE, AFP, PSA, Ca-19-9) were negative. The kidney biopsy revealed "Extracapillar glomerulonephritis with Ig A depositis in DIF) and the patients received 6 monthly cyclophosphamide pulse.

Our second patient presented initially with acute appendicitis confirmed by the histological finding. There was no depositis in the DIF.

All the patients had complete recovery from their abdominal symptoms within approximately three weeks.

## Discussion

HSP usually presents with a classic tetrad of rash, polyarthralgias, abdominal pain and renal disease. The clinical presentation of HSP is more severe among adult and tends to be atypical: higher rate of severe and atypical GI problems and delayed renal complications [3].

Older patients showed much more severe renal and extra-renal manifestations than younger patients in which the joint manifestations appear to be the most frequent. However, the severe GI involvements are less common in adults than children [4,5]. The mechanism of GI involvement in HSP may include IgA deposits on small vessel wall and polymorphonuclear neutrophils (PMNs) infiltration around vessels [3].

It occurs in 50- 75% of adult patients [6].

In a recent retrospective study including 115 adults with the diagnosis of HSP, GI were found in 90 patients (78.2%). [7]; it was 24% in the study of Novak et all [8]

In a large Italian cohort of 250 patients, adults and children, with HSP, necrotic purpura was present in 96% of the cases, arthritis in 61% of the cases and GI involvement in 48% of cases. The main symptom was colicky abdominal pain. Bleeding occurred in 51% cases of GI involvement and was serious, requiring transfusion or surgery or leading to death in 11% of the cases [9].

In the retrospective study conducted by Chang and all, including 261 children with a diagnosis of HSP, 151 (58%) had abdominal pain and 46 (17.6%) had GI bleeding detected as positive stool occult blood. Seven patients had gross bloody stools [10].

When comparing 82 children and 20 adults with HSP, Uppal and all found that the frequency nausea, vomiting, malena/hematochezia and intussusception were the same in both groups. Adults had a higher frequency of diarrhea [11].

When studying 46 adults and 116 children with HSP, Blanco and all reported a higher incidence of renal (p < i0.001) and lower GI involvement at disease onset in adult HSP, but during the clinical course, GI involvement was the same in both age groups [12].

Trouiller and all tried to assess a score of severity proposed to use clinical and radiological (CT scan) severity scores to assess the usefulness and the efficacy of corticosteroid therapy. The authors conclude, by styding 34 cases of patients with HSP, 68% of them with GI involvement, that these results suggest that corticosteroids may reduce abdominal symptoms of HSP in adults with clinically severe disease [13].

In rare cases, GI involvements revealed HSP, as in our 3 patients. It was reported by Farah [14] and Moles [15]. Al Toma and all described a case of a 40- year's old man who presented with terminal ileitis complicated by thrombotic microangiopathy revealing HSP [16].

GI was the first manifestation of HSP in 11% of the patients of Trappani [17] and 8% of those of Coppo [8].

Another important retrospective study was performed by Chen and Kong including 161 patients; colicky abdominal pain was noticed in 98.1% and vomiting in 39.5% of the patients. In 25 (3%) of the reported cases, GI symptoms were manifested before skin rash [18].

The classic findings may consist in: Periumbilical colicky and abdominal pain (related to edema, bleeding), nausea, vomiting, diarrhea, constipation, abdominal distension which are usually mils and rarely severe enough to be confused with an acute abdomen leading to laparotomy. Rare GI manifestations may include: Intestinal perforation, ischemic vasculitis, intussusceptions, esophageal ulcers, pancreatitis, pseudomembranous colitis, extensive GI bloodloss requires hemodynamic monitoring, transfusion and occasionally emergent surgical intervention, appendicitis, massive bowel necrosis. Massive GI hemorrhage and grossly bloody or melanotic stools are respectively reported in 2% and 30% of the patients [13,19-22]. Simultaneous occurrence HSP and acute appendicitis is rarely observed, as in our second case [23].

Mucosal lesions develop anywhere within the GI tract. Diffuse mucosal redness, small ring-like petechiae and hemorrhagic erosions are characteristic endoscopic findings. The small intestine is considered to be the most frequently affected site. Duodenal involvement was more prominent in the second part of the duodenum than in the bulb [24].

Imaging studies consisting in abdominal X -ray film, abdominal echography, and abdominal CT, and stool occult blood tests are important examinations [10]. Laparotomy which is performed in approximately 10% is useful when esophagogastroduodenoscopy, colonoscopy and small bowel barium radiogram show normal results. Videocapsule endoscopy represents a unique improvement in the investigation of intestinal diseases, allowing an excellent visualization of the mucosa of the small bowel [25].

Malignancy also has been reported as a rare causative factor of HSP [26]. The recent retrospective study conducted by Mitsui and all found 53 cases of malignant tumors [27]. Our first patient had colic dysplasia. No previous association has been reported so far.

Helicobacter pylori (Hp) infection may be associated with the development and relapse of HSP with gastrointestinal involvement among children; Wang found that 21 of 36 HSP patients with GI manifestations were confirmed with Hp infection (58.3%). Among those patients, the relapsed patients had a Hp positive rate of 81.3% (13/16), which was significantly higher than the newly diagnosed patients (45.0%, 9/20; P < 0.05). Two of our patients had Hp infection at the moment of the diagnosis [28].

When studying 11 adult patients with HSP and 20 healthy subjects, Novak concluded that IgG antibodies to Hp may be present mostly in acute HSP, while IgA antibodies may be involved in sustaining GI symptoms underlying the chronic phase of the disease [29].

The cutaneous pathology of Hp is far from being clear, but it is speculated that the systemic effects may involve increased mucosal permeability to alimentary antigens, immunomodulation, an autoimmune mechanism or the impairment of vascular integrity [30].

Recently, polymorphism at codon 469 of the intercellular adhesion molecule-1 (ICAM-1) locus has been associated with protection against severe gastrointestinal complications in HSP [31].

In most cases, HSP spontaneously disappears without treatment. The use of corticosteroids is controversial and usually reserved for severe systemic manifestations [32].

Steroid treatment makes improvement in the GI symptoms at an early stage but does not prevent the recurrence of abdominal pain attacks. Steroids decrease the intestinal wall edema and thus the symptoms improve or disappear.

In a Randomized, Double-Blind, Placebo-Controlled Trial, including 171 patients with HSP, Prednisone was effective in reducing abdominal pain (p = 0,028); the incidence of severe abdominal pain necessitating hospital admission was greater in the placebo group than in the prednisone group [33]. These data are in keeping with those of Peru and all; these authors conclude that the steroid treatment given to HSP patients with GI manifestations might be helpful to prevent probable complications such as bleeding and intussusceptions [34].

The use of gastric acid secretion inhibitors are also very beneficial on GI involvement.

Colchicina can be used in the treatment of HSP [35,36]. The rationale for using colchicine to treat HSP is based on the fact that colchicine perturbs microtubule function of the polymorphonuclear cell cytoskeleton [37]. This results in inhibition of polymorphonuclear cell migration to the site of inflammation.

## Conclusion

GI involvements are frequent in HSP. The diagnosis is more difficult when they precede the other manifestations and especially the cutaneous rash. Treatments are still controversial.

## Authors' contributions

AH: Wrote the manuscript; WM: Done the gastric exploration; OH: participate to the writing of the manuscript; LN: conducted the genetic study; RM: participate to the writing of the manuscript; MS: Participate to the coordination. All authors read and approved the final manuscript.

## Competing interests

The authors declare that they have no competing interests.
